# Effect of Preexisting Asthma on the Risk of ICU Admission, Intubation, and Death from COVID-19: A Systematic Review and Meta-Analysis

**DOI:** 10.1155/2022/8508489

**Published:** 2022-06-06

**Authors:** Abhinav Bhattarai, Garima Dhakal, Sangam Shah, Aastha Subedi, Sanjit Kumar Sah, Shyam Kumar Mishra

**Affiliations:** ^1^Maharajgunj Medical Campus, Institute of Medicine, Tribhuvan University, Maharajgunj 44600, Nepal; ^2^Central Department of Public Health, Institute of Medicine, Tribhuvan University, Maharajgunj 44600, Nepal; ^3^Tribhuvan University Teaching Hospital, Maharajgunj 44600, Nepal; ^4^Department of Microbiology, Maharajgunj Medical Campus, Institute of Medicine, Tribhuvan University, Maharajgunj 44600, Nepal

## Abstract

**Background:**

The Centers for Disease Control and Prevention (CDC) identifies asthma as a comorbidity in COVID-19 that increases the risk of severity and death. However, research has shown that asthma is not associated with increased severity and death, thus making the consequences of asthma in COVID-19 unclear.

**Methods:**

We searched the electronic databases PubMed, WHO COVID-19 database, and Taylor and Francis Online for studies that compared the medical outcomes of COVID-19 between patients with and without asthma, from the emergence of SARS-CoV-2 in December 2019 to the 3^rd^ of September 2021, excluded duplicates, reviews, editorials, and case reports, and screened the titles, abstracts, and full texts. The quality of the included studies was assessed using the Newcastle–Ottawa Scale (NOS) for nonrandomized studies. Rates of intensive care unit (ICU) admission, intubation, and death among patients with and without asthma were compiled and meta-analysis was conducted using a random-effects model.

**Results:**

Nineteen studies with a total of 289,449 participants met the inclusion criteria. COVID-19 patients with asthma had no significant association with increased risk of ICU admission, intubation, and death as compared with those without asthma ((odds ratio (OR) = 1.25, confidence interval (CI) = 0.90–1.74, *I*^2^ = 82%, *X*^2^ = 55.13, *p* < 0.01), (OR = 0.89, CI = 0.59–1.34, *I*^2^ = 91%, *X*^2^ = 110.82, *p* < 0.01), and (OR = 0.90, 95% CI = 0.63–1.27, *I*^2^ = 88%, *X*^2^ = 146.96, *p* < 0.01)), respectively.

**Conclusion:**

Preexisting asthma did not significantly increase the risk of poorer prognosis and death from COVID-19.

## 1. Introduction

The first case of the coronavirus disease-2019 (COVID-19) was reported from Wuhan, China, in December 2019. And as of now, 3^rd^ December 2021, which is 2 years from the emergence of SARS-CoV-2, over 230,000,000 cases have been diagnosed and over 4,750,000 deaths have been reported in over 200 countries [1]. A variety of risk factors are linked to having poorer medical prognosis and outcomes in COVID-19, some of which are old age, obesity, male sex, and comorbidities such as hypertension, chronic kidney disease, diabetes, asthma, chronic obstructive pulmonary disease (COPD), immunodeficiency, malignancies, and cardiovascular diseases [2].

Asthma is one of the most common chronic respiratory diseases in the world. The Centers for Disease Control and Prevention (CDC) and the World Health Organization (WHO) point out asthma as a comorbidity, and subjects with asthma are more likely to develop and die from severe complications in COVID-19 [2, 3]. However, from recent research, findings suggest poorer COVID-19 outcomes in patients with asthma as well as no association of asthma with poor prognosis and outcomes making the true consequences of asthma in COVID-19 susceptibility unclear.

The possible poor outcome might exist due to an interplay between the pathobiology of SARS-CoV-2 and asthma. Like other respiratory viruses, SARS-CoV-2 has a potential role in asthma exacerbation, making the infection even worse [4]. SARS-CoV-2's entry into the lung tissue, facilitated by the angiotensin converting enzyme-2 (ACE-2), creates exaggerated inflammatory response and widespread dissemination of the virus throughout the lung parenchyma, producing pneumonia [5]. In people with asthma, whose airways are already narrowed and mucus-filled, pneumonia may lead to worse outcomes [6]. To circumvent this, the pulmonary production of protective interferons (IFNs), usually IFN-*α*, IFN-*β*, and IFN-*λ*, are required [7]. Different studies report that the interferon production is significantly compromised in these patients which could further impede the immunity in seizing the viral spread, therefore worsening the outcomes of COVID-19 [8–10]. Contrary to this, poorer outcomes may be avoided by asthma due to the downregulation of ACE-2, the gateway of SARS-CoV-2 entry into lung tissue [11].

During this time of the COVID-19 pandemic where the virus is affecting the respiratory system, asthma patients are more concerned for their safety and recovery if infected [12]. Most of the systematic reviews to date have reported risks without specification to confounding factors, interventions, or specific outcomes which have made the true risk and implications more difficult to conceptualize. According to the statistics, the risk of asthmatics being admitted to the ICU, if hospitalized due to COVID-19, is statistically insignificant as compared to individuals who do not have asthma. Therefore, we conducted a systematic review and meta-analysis of the studies that reported the medical prognosis and outcomes of COVID-19 patients in three specific variables, intensive care unit (ICU) admission, intubation, and mortality, which can aid in assuring the people and healthcare systems the true risks of poorer outcomes in COVID-19.

## 2. Search Strategy and Study Selection

The systematic review was performed in accordance with Preferred Reporting Items for Systematic Reviews and Meta-Analyses (PRISMA) guidelines [13] and registered in PROSPERO (CRD42021281934).

A comprehensive search was performed on online databases PubMed, WHO COVID-19 database, and Taylor and Francis Online for all relevant articles published from the emergence of COVID-19 in December 2019 to the 3^rd^ of September 2021. The search terms used were “COVID-19,” “coronavirus,” “SARS-CoV-2,” “severity,” “mortality,” “severity,” “risk,” “asthma,” and “asthmatic” connected by “OR” and “AND” Boolean operators. The full search string is (COVID-19 OR coronavirus OR SARS-CoV-2) AND (severe^*∗*^ OR risk OR factor OR mortality OR critical) AND (asthma OR asthmatic). Thus, retrieved articles were then exported on an Excel® sheet, and duplicates were removed. The studies were subjected to screening by title and abstract, later followed by full-text screening by two investigators (AB and GD). Any discrepancies between the authors in the study selection process were solved through discussion and consensus from the supervisors (SS and SKM).

### 2.1. Eligibility Criteria

Studies were included if they fulfilled the following criteria:

1. The study population size was greater than 100
2. Participants were diagnosed with COVID-19 by a positive SARS-CoV-2 RT-PCR (severe acute respiratory syndrome coronavirus-2 reverse transcriptase polymerase chain reaction)
3. The population was categorized into asthmatic and nonasthmatic or at least specified asthma in the study
4. Data were provided on the medical outcomes of COVID-19 hospitalized patients including ICU admission, intubation, and death

The following criteria were the exclusion criteria of our study:

1. Studies that did not show the prevalence or did not specify asthma in hospitalized COVID-19 patients
2. Articles whose full text was not accessible in English
3. Studies that only included the pediatric population, geriatric population, or pregnant women
4. Other articles such as case reports, editorials, review articles, case series, and perspectives

### 2.2. Data Extraction and Quality Assessment

Two investigators AB and GD manually extracted the data into a prespecified data collection form consisting of the following variables: author, study design, the country where the study was conducted, sample size, age, sex, events of ICU admission, events of intubation, and events of death. The extracted data were tabulated in Microsoft Excel® 2019 version (Microsoft Corporation).

For assessing the quality of the studies, the Newcastle–Ottawa Scale (NOS) for nonrandomized studies was used. The NOS is a scoring system of studies based on the fulfillment of a researcher's aim in 3 specific domains, selection, comparability, and outcome, in which stars are assigned as points [14]. The scoring was performed by two investigators (AB and GD). The total score was then assessed where 7–9 was considered a high-quality study with a low risk of bias, 4–6 as high risk of bias, and 0–3 as very high risk of bias. Any discrepancies during the risk of bias assessment were solved by the consensus of supervisor SS.

### 2.3. Outcomes of Interest

The primary outcome of interest was to assess whether asthmatic patients with COVID-19 had an increased risk of ICU admission, intubation, and mortality when compared to nonasthmatic patients with COVID-19.

### 2.4. Statistical Analysis

Meta-analysis of the extracted data was carried out by two investigators (AB and AS) using software *R* version 4.1.3. Effect sizes on events of ICU admission, intubation, and death were reported as odds ratio (OR) and were compared between the two populations: asthmatic and nonasthmatic under the following headings:

1. Risk of ICU admission
2. Risk of intubation
3. Risk of mortality

Heterogeneity was assessed with Cochrane's *Q* test and *I*^2^ statistic where values < 25%, 25%–50%, and >50% represented low, moderate, and high heterogeneity, respectively. Considering the high heterogeneity between the studies, a random-effects model was adopted to estimate the pooled OR under 95% confidence interval (CI), and forest plots were created for interpretation. “*tau*” was estimated using the DerSimonian and Laird method. In addition, sensitivity analysis was conducted to evaluate the stability of the outcome and was performed by excluding one study at a time. *p* < 0.05 was considered statistically significant.

## 3. Results

### 3.1. Study Selection

**Figure 1:**
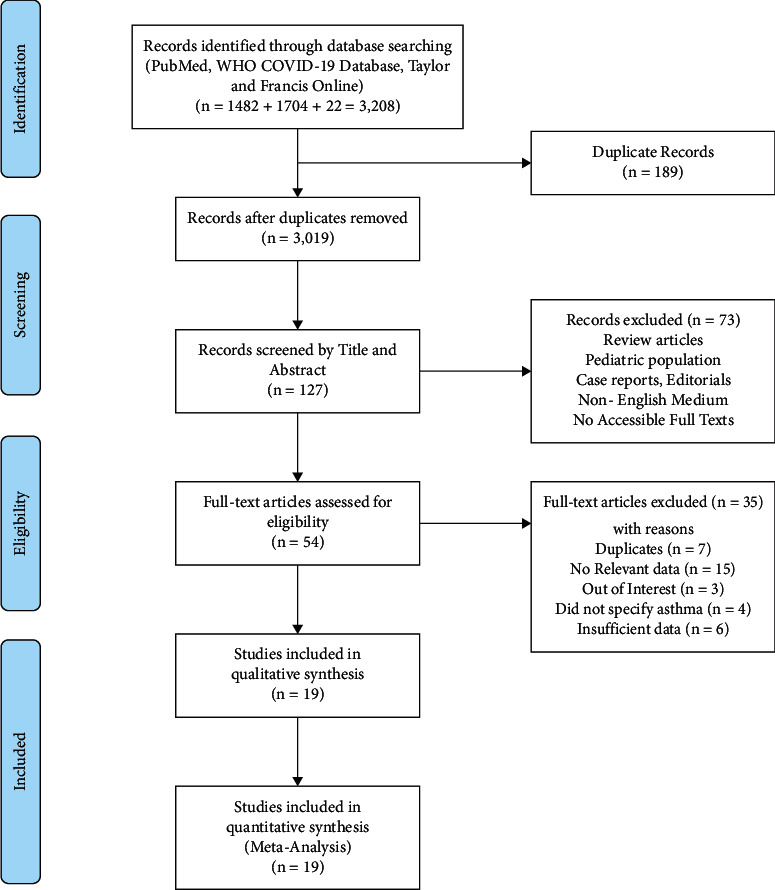
PRISMA flow diagram.

The literature search retrieved 3,208 articles. After the duplicates were removed, the remaining articles were screened by title and abstract followed by full-text screening, and finally, 19 studies that fulfilled the inclusion criteria were included for qualitative as well as quantitative synthesis. The full study selection is displayed in Figure 1.

### 3.2. Study Characteristics

The descriptive characteristics of the included studies are displayed in Table 1. There were a total of 289,449 participants in the included studies, and almost all of them were older than 15 years. COVID-19 status in all was confirmed by SARS-Cov-2 RT-PCR. Of the nineteen included studies, eight [15, 16, 18, 24, 27, 29, 30, and 33] were from North America, five [17, 22, 26, 28, and 31] from Asia, five [19–21, 23, and 25] from Europe, and one [32] from South America. Eighteen [ and 15– –33] were cohort studies, while one [21] was cross-sectional. All the studies reported data on ICU admission or intubation or death or all among the hospitalized COVID-19 patients with and without asthma.

### 3.3. Quality Assessment

**Figure 2:**
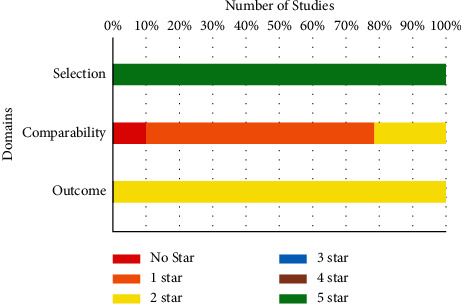
Quality assessment of studies.

The result of the quality assessment among the studies is shown in Table 2. All the studies were high-quality ones ranging from 7 to 9. The mean score was 8.10.  Figure 2 summarizes the quality assessment of the studies. Sensitivity analyses that excluding each study one at a time had no significant effect on the overall estimates.

### 3.4. Clinical Outcomes of Included Studies

The outcomes of ICU admission, intubation, and death in each study are shown in Table 3. The rates in each study varied but correlated.

## 4. Meta-Analysis

### 4.1. Risk of ICU Admission

**Figure 3:**
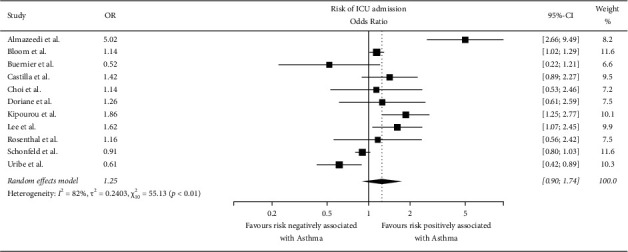
Forest plot showing the risk of ICU admission.

The number of studies that reported the ICU admission rates was 11. The pooled odds ratio (OR) was 1.25 (*I*^2^ = 82%, *X*^2^ = 55.13, *p* < 0.01, and CI = 0.90–1.74) (Figure 3). According to the statistics, the risk of being admitted to the ICU if hospitalized due to COVID-19 is statistically insignificant compared to individuals who do not have asthma.

### 4.2. Risk of Intubation

**Figure 4:**
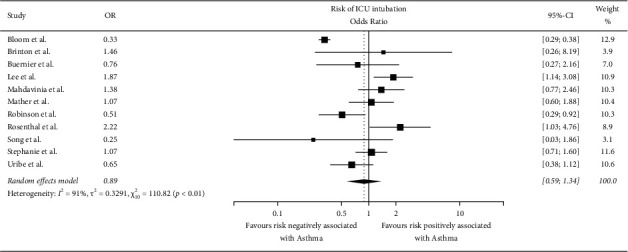
Forest plot showing risk of intubation.

The number of studies that reported the intubation rates was 11. The pooled odds ratio (OR) was 0.89 (*I*^2^ = 91%, *X*^2^ = 110.82, *p* < 0.01, and CI = 0.59–1.34) (Figure 4). The statistics showed that although the *p* value is less than 0.01, asthma did not significantly increase the risk of being intubated due to COVID-19 due to lower odds ratio.

### 4.3. Risk of Mortality

**Figure 5:**
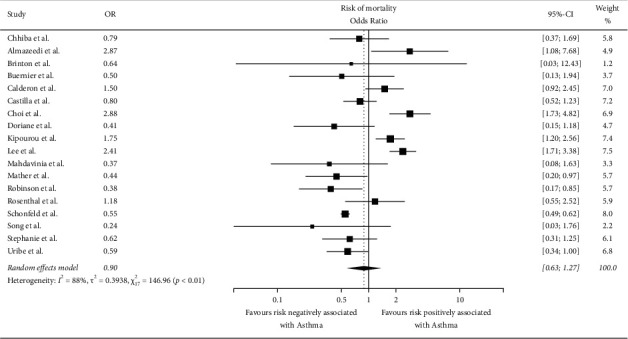
Forest plot showing the risk of mortality.

The number of studies that reported the mortality rates was 18. The pooled odds ratio (OR) was 0.90 (*I*^2^ = 88%, *X*^2^ = 146.96, *p* < 0.01, and CI = 0.63–1.27) (Figure 5). The statistics showed that asthma in hospitalized patients was not statistically associated with an increased risk of mortality due to COVID-19 due to lower odds ratio.

## 5. Discussion

Our study aimed at evaluating the risk of poor prognosis in COVID-19 in patients with preexisting asthma. Few studies reported in favor of poor prognosis of COVID-19 in asthmatic patients [18, 22, 26, 28, 31]. However, other scientific observations have reported conflicting results [15–17, 19–21, 23–25, 27, 29, 30, 32, 33]. Our meta-analysis did not identify asthma as a significant risk factor in producing high severity and mortality from SARS-CoV-2. Asthma is a disease that has been shown to be acutely exacerbated by respiratory viral infections [34], but it is unclear on which viruses are involved and how severe the exacerbation is.

Branco et al. [35] have explained the possible role of angiotensin-converting enzyme-2 (ACE-2) receptor expression behind the lowered risk of severity in asthma. SARS-CoV-2 gets access to the host cell when its spike protein binds to the ACE-2. ACE-2 is largely expressed in the lungs and less in other organs such as the heart and the kidneys [36], and this can explain the respiratory involvement in SARS-CoV-2 infection. Theoretically, it seems that the susceptibility of COVID-19 also depends on ACE-2 expression, and the susceptibility can be altered by either upregulation or downregulation of the receptor. Type I and Type II interferons (IFNs) are known to upregulate ACE-2 expression. However, the damaged IFN responses in the asthmatic status confer to downregulation of the receptor, hence limiting the invasion of SARS-CoV-2 into the host cell [37], and this could probably explain the lowered risk. In contrast to this fact, Kaur et al. [38] showed that ACE-2 possesses a protective effect in lung pathology and its downregulation might compromise its protective effect. This suggests that ACE-2 downregulation in asthmatics is not the sole cause behind the protective mechanism, and in contradiction, it can worsen any lung pathology. According to Jeon et al. [39], the inhaled corticosteroid (ICS) therapy given to asthmatics might have beneficial effects on COVID-19, thus reducing the susceptibility.

The nature of asthma either allergic or nonallergic is also found to be involved in minimizing the risks, and the allergic nature seemed to be in favor of minimal risk [40]. The Th phenotype of asthma may have significantly different COVID-19 prognosis as shown by Bloom et al. [41]. People with moderate to severe forms of asthma are more likely to have a poorer prognosis than people with mild forms [42]. Although vaccination programs have been going on, COVID-19 cases are still rising all across the globe and also is the prevalence of asthma due to increased air pollution. Therefore, the actual mechanism is essential to be established from more observations, studies, and analyses so that COVID-19 and asthma can be made to discontinue going side by side in causing more fatalities.

The main strength of our study is that a large population is included from follow-up cohort studies to cross-sectional studies, and the pooled outcome can apply to the general population. There are a few limitations of our study: First is that the type of asthma, allergic or nonallergic, and status could not be accessed. Secondly, the asthma severity status of the patient was not taken into consideration. Thirdly, we could not access the ICS therapy status of the population. Fourthly, the publication bias of the studies was not accessed, and lastly, insufficiency in the analysis might be created due to the potential omission of studies that might have valuable data.

## 6. Conclusion

Asthma was not significantly associated with a higher risk of ICU admission, intubation, and risk of mortality. However, the focus on COVID-19 and asthma should not be withdrawn unless more studies are conducted which provide stronger evidence that asthma is not a significant risk factor in the case of COVID-19. Individuals with asthma, to an extent, can be assured towards their well-being during the pandemic but should never compromise on safety and preventive measures.

## Figures and Tables

**Table 1:** Brief description of the included studies.

S. no.	Study	Study design	Study country	Sample size	Age groups	Sex (M : F)	Available variables	Result of the study
1	Chhiba et al. [15]	Retrospective cohort	United States of America	1542	<40, 40–70, >70	9 : 10	Mortality	Statistically no association with increased risk of mortality due to COVID-19 in asthmatics
2	Stephanie et al. [16]	Retrospective cohort	United States of America	1298	51	15 : 10	Intubation, mortality	Similar association with intubation and statistically lower risk of mortality in asthma
3	Song et al. [17]	Retrospective cohort	Wuhan, China	961	63 (49,70)	11 : 10	Intubation, mortality	The risk of intubation and mortality were statistically insignificant in asthmatics
4	Mahdavinia et al. [18]	Cohort	United States of America	935	18–65	8 : 10	Intubation, mortality	Statistically, a higher risk of being intubated but a lower risk of mortality in asthma
5	Beurnier et al. [19]	Prospective cohort	France	112	54 (42,67)	3 : 7	ICU admission, intubation, death	No significant association with increased risk of ICU admission, intubation, and mortality in asthma
6	Doriane et al. [20]	Retrospective cohort	Belgium	596	53 ± 18	9 : 10	ICU admission, mortality	Insignificant association between asthma and risk of ICU admission and mortality
7	Calderon et al. [21]	Cross-sectional	Spain	6310	55 ± 20	4 : 10	Mortality	Similar association with mortality
8	Choi et al. [22]	Retrospective cohort	Korea	7590	55.5(41,71)	6 : 10	ICU admission, mortality	Statistically higher association with ICU admission and mortality from COVID-19 in asthma.
9	Bloom et al. [23]	Prospective cohort	United Kingdom	8950	16–49	13 : 10	ICU admission, intubation	No association with increased risk of ICU admission and intubation due to COVID-19 in asthma
10	Robinson et al. [24]	Cohort	United States of America	3248	≥18	3 : 10	Intubation, mortality	Lower association with risk of intubation and mortality in asthma
11	Castilla et al. [25]	Prospective cohort	Spain	35387	<15, 15–65, >65	9 : 10	ICU admission, mortality	Insignificant association with ICU admission and mortality in asthma
12	Almazeedi et al. [26]	Retrospective cohort	Kuwait	1096	41(25–57)	42 : 10	ICU admission, mortality	Significant association with ICU admission and mortality in asthma
13	Mather et al. [27]	Retrospective cohort	United States of America	1045	NR	NR	Intubation, mortality	Insignificant association with increased risk of intubation in asthma
14	Lee et al. [28]	Retrospective cohort	Korea	7272	>20	6 : 10	ICU admission, intubation, mortality	Significantly higher risk of ICU admission, intubation, and mortality in asthma
15	Uribe et al. [29]	Cohort	United States of America	961	66(52,78)	9 : 10	ICU admission, intubation, mortality	Lower rates of ICU admission, intubation, and mortality in asthma. Insignificant statistical association
16	Brinton et al. [30]	Retrospective cohort	United States of America	345	49 ± 6	NR	Intubation, mortality	Equal association with risk of intubation and mortality between asthmatic and nonasthmatic patients
17	Kipourou et al. [31]	Prospective cohort	Kuwait	3995	41 ± 7	24 : 10	ICU admission, mortality	Statistically significant risk of ICU admission and mortality in asthma
18	Schonfeld et al. [32]	Retrospective cohort	Argentina	207079	41(2,55)	9 : 10	ICU admission, mortality	Similar association of risk of ICU admission mortality in asthma
19	Rosenthal et al. [33]	Retrospective cohort	United States of America	727	46.61	NR	ICU admission, intubation, mortality	Higher association with risk of ICU admission, intubation, and mortality in asthma

Age is expressed in median (IQR) or mean (SD) or range. SARS-Cov-2 RT-PCR: severe acute respiratory syndrome coronavirus-2 reverse transcriptase polymerase chain reaction.

**Table 2:** Newcastle–Ottawa Scale (NOS) for quality assessment of nonrandomized studies.

Author	Selection				Comparability	Outcome		Total score
	Representativenessof the sample	Samplesize	Nonrespondents/recruitment rate	Ascertainmentof exposure	The total population categorizedinto with and without asthma anddata adjusted for ICUadmission, intubation, and death	Assessmentof outcome	Adequacy offollow-up	
Chhiba et al. [15]	1	1	1	2	0	1	1	7
Stephanie et al. [16]	1	1	1	2	1	1	1	8
Song et al. [17]	1	1	1	2	1	1	1	8
Mahdavinia et al. [18]	1	1	1	2	1	1	1	8
Beurnier et al. [19]	1	1	1	2	2	1	1	9
Doriane et al. [20]	1	1	1	2	1	1	1	8
Calderon et al. [21]	1	1	1	2	0	1	1	7
Choi et al. [22]	1	1	1	2	1	1	1	8
Bloom et al. [23]	1	1	1	2	1	1	1	8
Robinson et al. [24]	1	1	1	2	1	1	1	8
Castilla et al. [25]	1	1	1	2	1	1	1	8
Almazeedi et al. [26]	1	1	1	2	1	1	1	8
Mather et al. [27]	1	1	1	2	1	1	1	8
Lee et al. [28]	1	1	1	2	2	1	1	9
Uribe et al. [29]	1	1	1	2	2	1	1	9
Brinton et al. [30]	1	1	1	2	1	1	1	8
Kipourou et al. [31]	1	1	1	2	1	1	1	8
Schonfeld et al. [32]	1	1	1	2	1	1	1	8
Rosenthal et al. [33]	1	1	1	2	2	1	1	9

**Table 3:** Data on ICU admission, intubation, and death in patients with and without asthma in the studies.

S. no.	Study	ICU admission, *n* (%)		Intubation, *n* (%)		Death, *n* (%)	
		Asthma	Nonasthma	Asthma	Nonasthma	Asthma	Nonasthma
1	Chhiba et al. [15]	NR	NR	NR	NR	8 (3.6)	64 (4.9)
2	Stephanie et al. [16]	NR	NR	34 (21)	231 (20)	9 (6)	101 (9)
3	Song et al. [17]	NR	NR	1 (4.6)	141 (15.4)	1 (4.6)	148 (16.1)
4	Mahdavinia et al. [18]	NR	NR	23 (9.7)	56 (8.3)	2 (1.1)	16 (3.0)
5	Beurnier et al. [19]	11 (30)	33 (44)	6 (16.2)	15 (20)	3 (8.1)	11 (14.6)
6	Doriane et al. [20]	10 (17.5)	78 (14.5)	NR	NR	4 (7)	83 (15.4)
7	Calderon et al. [21]	NR	NR	NR	NR	21(3.64)	229 (3.99)
8	Choi et al. [22]	7 (3.2)	208 (2.8)	NR	NR	17 (7.8)	210 (2.8)
9	Bloom et al. [23]	451 (24.1)	1542 (21.7)	269 (14.4)	953 (13.4)	NR	NR
10	Robinson et al. [24]	NR	NR	15 (3)	107 (4)	7 (1)	69 (3)
11	Castilla et al. [25]	23 (0.98)	223 (0.67)	NR	NR	28 (1.2)	438 (1.3)
12	Almazeedi et al. [26]	18 (11.6)	24 (2.5)	NR	NR	6 (3.8)	13 (1.3)
13	Mather et al. [27]	NR	NR	16 (18.2)	165 (17.2)	7 (8)	157 (16.4)
14	Lee et al. [28]	27 (3.9)	163 (2.4)	19 (2.7)	99 (1.5)	44 (6.4)	183 (2.7)
15	Uribe et al. [29]	47 (29.9)	330 (41)	17 (10.8)	126 (15.6)	17 (10.8)	138 (17.1)
16	Brinton et al. [30]	NR	NR	2 (8.3)	17 (5.2)	0 (0)	7 (2.8)
17	Kipourou et al. [31]	31 (13.2)	284 (7.6)	NR	NR	35 (14.9)	341 (9)
18	Schonfeld et al. [32]	296 (2.3)	5383 (2.7)	NR	NR	396 (3.1)	10517 (5.4)
19	Rosenthal et al. [33]	11 (10.5)	57 (9.1)	11 (10.5)	33 (5.3)	10 (9.5)	51 (8.2)

NR: not reported.

## Data Availability

All the required information is included within the article.
